# Pharmacological Characterization of the Native Store-Operated Calcium Channels of Cortical Neurons from Embryonic Mouse Brain

**DOI:** 10.3389/fphar.2016.00486

**Published:** 2016-12-12

**Authors:** Sylvain Chauvet, Louis Jarvis, Mireille Chevallet, Niroj Shrestha, Klaus Groschner, Alexandre Bouron

**Affiliations:** ^1^UMR CNRS 5249, Commissariat à l’Énergie Atomique et aux Énergies AlternativesGrenoble, France; ^2^Université Grenoble AlpesGrenoble, France; ^3^Institut de Biosciences et Biotechnologies de Grenoble – Laboratoire de Chimie et Biologie des MétauxGrenoble, France; ^4^Institute of Biophysics, Medical University of GrazGraz, Austria

**Keywords:** store-operated channels, calcium, Orai channels, neurons, cortex

## Abstract

In the murine brain, the first post-mitotic cortical neurons formed during embryogenesis express store-operated channels (SOCs) sensitive to Pyr3, initially proposed as a blocker of the transient receptor potential channel of C type 3 (TRPC3 channel). However, Pyr3 does not discriminate between Orai and TRPC3 channels, questioning the contribution of TRPC3 in SOCs. This study was undertaken to clarify the molecular identity and the pharmacological profile of native SOCs from E13 cortical neurons. The mRNA expression of STIM1-2 and Orai1-3 was assessed by quantitative reverse transcription polymerase chain reaction. E13 cortical neurons expressed STIM1-2 mRNAs, with STIM2 being the predominant isoform. Only transcripts of Orai2 were found but no Orai1 and Orai3 mRNAs. Blockers of Orai and TRPC channels (Pyr6, Pyr10, EVP4593, SAR7334, and GSK-7975A) were used to further characterize the endogenous SOCs. Their activity was recorded using the fluorescent Ca^2+^ probe Fluo-4. Cortical SOCs were sensitive to the Orai blockers Pyr6 and GSK-7975A, as well as to EVP4593, zinc, copper, and gadolinium ions, the latter one being the most potent SOCs blocker tested (IC_50_ ∼10 nM). SOCs were insensitive to the TRPC channel blockers Pyr10 and SAR7334. In addition, preventing mitochondrial Ca^2+^ uptake inhibited SOCs which were unaffected by inhibitors of the Ca^2+^-independent phospholipase A_2_. Altogether, Orai2 channels are present at the beginning of the embryonic murine cortico-genesis and form the core component of native SOCs in the immature cortex. This Ca^2+^ route is likely to play a role in the formation of the brain cortex.

## Introduction

Store-operated channels (SOCs) are Ca^2+^-conducting channels of the plasma membrane (PM) that open in response to the emptying of a pool of Ca^2+^ stored in the endoplasmic reticulum (ER). SOCs mediate a prominent Ca^2+^ signaling process called store-operated Ca^2+^ entry (SOCE), initially named capacitative Ca^2+^ entry (CCE; Putney, 1986). Although neurons express numerous types of Ca^2+^-conducting channels, which could make this pathway redundant or even unnecessary, they nevertheless exhibit a SOCE. The first studies providing experimental evidence for the existence of a putative neuronal SOCE appeared nearly 25 years ago. They were carried out on cells lines (N1E-115 and PC12 cells) and showed that the passive leakage of Ca^2+^ out of the ER induced by the sarco/endoplasmic Ca^2+^ ATPase (SERCA) inhibitor thapsigargin is associated with an influx of Ca^2+^ (Takemura et al., 1991; Clementi et al., 1992; Mathes and Thompson, 1994). Since then, SOCE has been observed in diverse neuronal preparations including cultured dorsal root ganglion neurons (Usachev and Thayer, 1999), freshly dissociated olfactory receptor neurons (Zufall et al., 2000), cultured hippocampal (Bouron, 2000), and cortical neurons (Prothero et al., 2000; Yoo et al., 2000).

The current molecular description of SOCE proposes that SOCs consist of Orai channels recruited by STIM (Stromal interaction molecules), a family of Ca^2+^ sensors of the ER. To date, two STIM (STIM1-2) and three Orai (Orai1-3) isoforms are known (Hoth and Niemeyer, 2013; Prakriya and Lewis, 2015). But whether this STIM/Orai complex is necessary and sufficient to fully reconstitute native SOCs is still an open question. Indeed, some authors propose a more complex scenario with additional actors such as transient receptor potential channels of C type (TRPC channels). According to this latter view, STIM, Orai, and TRPC would be necessary to generate a SOCE (Birnbaumer, 2009; Lee et al., 2010; Cheng et al., 2013).

In the murine brain, the onset of cortico-neurogenesis occurs at ∼E12 (Takahashi et al., 1995). Since Ca^2+^ signaling plays a critical role in cortical development, it is important to understand how the first post-mitotic neurons acquire this essential cation. Ca^2+^ imaging experiments revealed that murine E13 cortical neurons display a SOCE (Bouron et al., 2005; Gibon et al., 2010) that is inhibited by Pyr3 with an IC_50_ of ∼0.5 μM (Gibon et al., 2010), a pyrazole derivative originally described as a selective TRPC3 channels inhibitor (Kiyonaka et al., 2009). This finding suggested that TRPC3 channels could be critical components of native SOCs in cortical neurons. However, subsequent studies found that Pyr3 does not discriminate between TRPC3 and Orai1 since it blocks these channels with the same potency (Schleifer et al., 2012), leaving some doubt on the contribution of TRPC3 in SOCE of embryonic cortical neurons. Therefore, the following study was undertaken to better characterize the molecular identity as well as the pharmacological profile of native SOCs from E13 cortical neurons.

## Materials and Methods

### Animal and Ethical Statement

The C57Bl6/J mice used in this study were from the Jackson Laboratory (USA). They were housed in temperature-controlled rooms under a 12 h light – 12 h dark cycle with *ad libitum* access to food and water. Two females were housed per cage whereas males were kept individually. In each instance, they were permanently exposed to an enriched environment in accordance with the Animal Welfare Committee of the CEA Grenoble. They were crossed once a week which allowed us to determine the age of the embryos. Pregnant mice were killed by cervical dislocation without prior anesthesia. The use of animals and all procedures were approved by the animal care committee of the CEA’s Life Sciences Division (CEtEA; # A14-006).

### Materials

SAR7334 and GSK-7975A were kind gifts from Sanofi-Aventis Deutschland and Dr. Malcolm Begg (GlaxoSmithKline), respectively. All the reagents were from Sigma-Aldrich (France, Austria) except for Fluo-4/AM, Fura-2/AM, and tissue culture media from Molecular Probes, Invitrogen (France, Austria).

### Cell Cultures of Cortical Neuron and RBL-2H3

Primary cultures of cortical neurons were prepared from E13 embryos according to experimental procedures described in Gibon et al. (2013) and Chauvet et al. (2015). Briefly, following removal of the meninges, ganglionic eminences, and olfactory bulbs, cerebral cortices of E13 mice (with the vaginal plug as E0) were isolated from 5 to 6 embryos and placed in 1 mL of an ice-cold Ca^2+^- and Mg^2+^-free Hank’s solution supplemented with 33 mM glucose, 4.2 mM NaHCO_3_, 10 mM HEPES, and 1% penicillin/streptomycin. Cortical cells were dissociated by mechanical trituration using a sterile Pasteur pipette. The cell suspension was filtered through a 40 μm cell strainer and isolated cells were plated at a density of ∼1.5 × 10^5^ cells/16 mm diameter glass coverslips before being placed into 35 mm diameter petri dishes containing 2 mL of a Neurobasal medium supplemented with B27 (2%) and glutamine (500 μM; Gibon et al., 2013). RBL-2H3 cells were cultured in Dulbecco’s Modified Eagle Medium (DMEM) containing 10% fetal bovine serum, 100 U/mL penicillin, and 100 μg/mL streptomycin. Cells were maintained in an incubator at 37°C in 5% CO_2_ atmosphere.

### Quantitative PCR

RNA from cortex and isolated neuron cells were isolated using Absolutely RNA miniprep kit (Agilent # 400800). RNA concentration was determined using a NanoDrop spectro photometer (ND-1000). Reverse transcription was performed with the Affinity script qPCR cDNA synthesis kit (Agilent # 600559) according to the manufacturer’s instructions. Gene specific primers for the different STIM and Orai murine genes were designed according to the primer software Primer-Blast. The designed primers are given in **Table 1**. Quantitative PCR was performed with Brilliant II SYBR green qPCR master mix1 (Agilent # 600828) using primers at 200 nM. PCR reaction mixtures (10 μl) were placed in a Cfx96 instrument (Bio-Rad) where they underwent the following cycling program, optimized for a 96-well block: 95°C for 15 min, immediately followed by 40 cycles of 10 s at 95°C and 30 s at 60°C. At the end, PCR products were dissociated by incubating for 1 min at 95°C and then 30 s at 55°C, followed by a ramp up to 95°C. PCR quality and specificity were verified by analyzing the dissociation curve. qRT-PCR reactions were run in triplicate, and quantification was performed using comparative regression (Cq determination mode). Quantitative PCR data were comparatively analyzed according to Pfaﬄ (2001) with 18S as reference gene. Results are expressed as relative quantity of mRNA.

**Table 1:** List of the primer sequences used for the quantitative PCR.

Gene	Forward primer sequence	Reverse primer sequence
Orai 1	cctggcgcaagctctactta	catcgctaccatggcgaagc
Orai 2	gtgggtctcatcttcgtggt	tcttcgatctcacggttgtg
Orai 3	tgcactgatggtctccacat	tgcactgatggtctccacat
STIM 1	ttgggcctcctctcttgact	gccacccacaccaataacga
STIM 2	aatcagcgaccgaagtcaca	ttatgaggtgggcgtgtcag
TRPC 1	aagcttttcttgctggcgtg	ctcccaagcacatctacgca

### Time-Lapse Ca^2+^ Imaging Experiments

The experimental setup was as described in Gibon et al. (2010), Tu et al. (2010), and Chauvet et al. (2015). Briefly, cells were incubated for 20 min at room temperature in the dark in a saline consisting of (in mM): 136 NaCl, 5 KCl, 2 CaCl_2_, 1 MgCl_2_, 10 HEPES, 10 glucose, pH 7.4 (NaOH) supplemented with 1.25 μM Fluo-4/AM. After dye loading, cells were rinsed twice and kept 10 min at room temperature in the dark before recording Fluo-4 responses. To this aim, the baseline Fluo-4 fluorescence was recorded for ≥1 min and averaged (F0). The results are expressed as F/F0 as a function of time, with F being the Fluo-4 fluorescence intensity. Each solution was prepared fresh each day from 1000× stock solutions so that the final concentration of DMSO never exceeded 0.1%. Images were acquired at room temperature at a frequency of 0.2 Hz with a CCD CoolSnap HQ2 camera (Princeton Instruments, Roper Scientific, France) placed on an inverted Axio Observer A1 microscope (Carl Zeiss, France) having a Fluar 40× oil immersion objective lens (1.3 NA, Carl Zeiss, France). A DG-4 wavelength switcher (Princeton Instruments, Roper Scientific, France) was used. The excitation light for Fluo-4 was filtered through a 470–495 nm excitation filter and the light was collected through a 525 nm filter. Data acquisition and off-line analysis were conducted with MetaFluor (version 7.0, Universal Imaging, Roper Scientific, France). All of the live cell Ca^2+^ imaging experiments were conducted on cultured neurons at 2–3 days *in vitro*.

Changes in [Ca^2+^]_i_ in RBL-2H3 cells were monitored using the Fura-2 technique as previously described (Schleifer et al., 2012). Cells grown on coverslips at a confluency of 50–60% were incubated in a saline consisting of (in mM): 136 NaCl, 5 KCl, 2 CaCl_2_, 1 MgCl_2_, 10 HEPES, 10 glucose, pH 7.4 (NaOH) supplemented with 2 μM Fura-2 AM for 40 min at room temperature in the dark. After the incubation period, cells were washed twice with saline and left to equilibrate for at least 20 min. The coverslip was then mounted in a perfusion chamber on an inverted microscope (Olympus IX71) and perfused with different solutions at room temperature. During the recordings using Live Acquisition 2.5 software (FEI, Germany), cells were excited alternately at 340 and 380 nm using an Oligochrome excitation system (FEI, Germany) and fluorescent images were captured at 510 nm every 1.2 s with an ORCA-03G digital CCD camera (Hamamatsu, Germany).

### Statistics

Data were expressed as mean ± standard error of the mean (SEM), with *n* being the number of cell bodies analyzed. Unless otherwise indicated, these cells originate from ≥3 distinct cell cultures. SigmaPlot (version 10.0, Systat Software) was used for plotting graphs and statistical analysis was performed using SigmaStat (version 3.5, Systat Software). The solid line shown in **Figure 4B** was obtained by a non-linear regression analysis using a sigmoid dose-response curve model. A Student’s *t*-test was used to assess the differences between two groups. Differences resulting in *p* < 0.05 were regarded as statistically significant.

## Results

### STIM and Orai mRNA Expression

**FIGURE 1:**
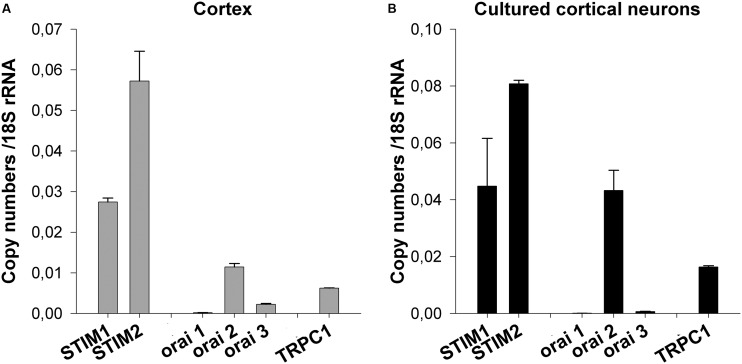
**Expression profile of STIM, Orai, and TRPC1 mRNAs.** mRNAs were extracted from E13 cortices and from cultured cortical neurons kept *in vitro* for 8 days. They were analyzed by quantitative real-time RT-PCR. The mRNA expression levels of STIM1, STIM2, Orai1, Orai2, Orai3, and TRPC1 are compared to 18S rRNA. Mean ± SEM of experiments conducted in triplicate. Profile of mRNA expression in total E13 brain cortices **(A)** and in dissociated E13 cultured neurons **(B)**.

First, the mRNA expression profile of STIM1-2 and Orai1-3 was assessed. mRNAs were extracted from E13 brain cortices and from E13 cortical neurons kept 8 days *in vitro* (DIV). For STIM, the pattern of mRNA expression was similar in each preparation (cortex and dissociated neurons) with STIM2 being the predominant STIM member (**Figure 1**). On the other hand, Orai1 mRNA was barely detectable whereas Orai3 mRNA was found at a low level in the cortex (**Figure 1A**). In cultured cortical neurons, mRNA expression for both Orai isoforms was hardly significant (**Figure 1B**) but the Orai2 mRNA was clearly expressed (**Figure 1**). The bar graph also shows the mRNA expression level of TRPC1, the most abundant TRPC isoform in the E13 cortex (Boisseau et al., 2009). It appears that the mRNAs of the SOC components STIM1-2 and Orai2 predominate over the TRPC1 mRNA.

**FIGURE 2:**
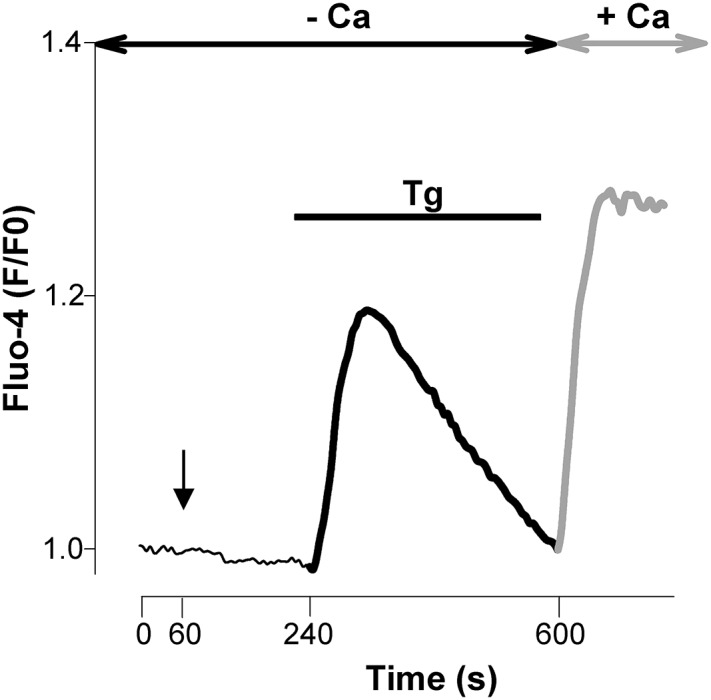
**Emptying intracellular pools of Ca^2+^ with thapsigargin controls an entry of Ca^2+^.** Unless otherwise indicated, the experimental protocol used throughout this study is illustrated in this figure. E13 cortical neurons loaded with Fluo-4 were maintained in a Ca^2+^-free saline. Thapsigargin (Tg, 200 nM) was added 240 s after the beginning of the recording and then washed away prior to the re-admission of Ca^2+^ (2 mM) into the recording saline medium (at time 600 s). Thick dark line: release of Ca^2+^ evoked by Tg; thick gray line: Ca^2+^ entry triggered by the emptying of the Ca^2+^ stores (SOCE). When used, blockers of channels were applied at time +60 s (vertical arrow) and remained present till the end of the recording. Antimycin-A was also added at time +60 s.

Next, Ca^2+^ imaging experiments were conducted to monitor the entry of Ca^2+^ through native SOCs of cultured E13 cortical neurons. This was done using the Ca^2+^ add back protocol illustrated in **Figure 2**: in order to elicit a SOCE, 200 nM thapsigargin (Tg) was applied from time 240 to 600 s on cells maintained in a Ca^2+^-free saline to deplete the ER Ca^2+^ stores prior to the introduction of 2 mM Ca^2+^ to the extracellular media at time 600 s. Tg has a high affinity for mammalian Ca^2+^ pumps of the SERCA but application of micromolar concentrations of this compound can elicit biological responses unrelated to SERCA pumps. Therefore Tg was used at a low concentration to avoid unwanted side effects.

### Neuronal SOCE and the STIM2 Inhibitor G418

The antibiotic G418 has been reported to be a potent STIM2 inhibitor preventing the activation of SOCs like Orai1 (Parvez et al., 2008). This makes G418 a valuable tool to assess STIM2 functions. In order to probe the relative contribution of STIM1-2 to native neuronal SOCs, experiments were conducted with this antibiotic. Cells were preincubated for >45 min at room temperature with 500 μg/mL G418. Under these conditions, G418 did not affect SOCE in embryonic cortical neurons (not shown).

### Neuronal SOCE and Blockers of Orai Channels

**FIGURE 3:**
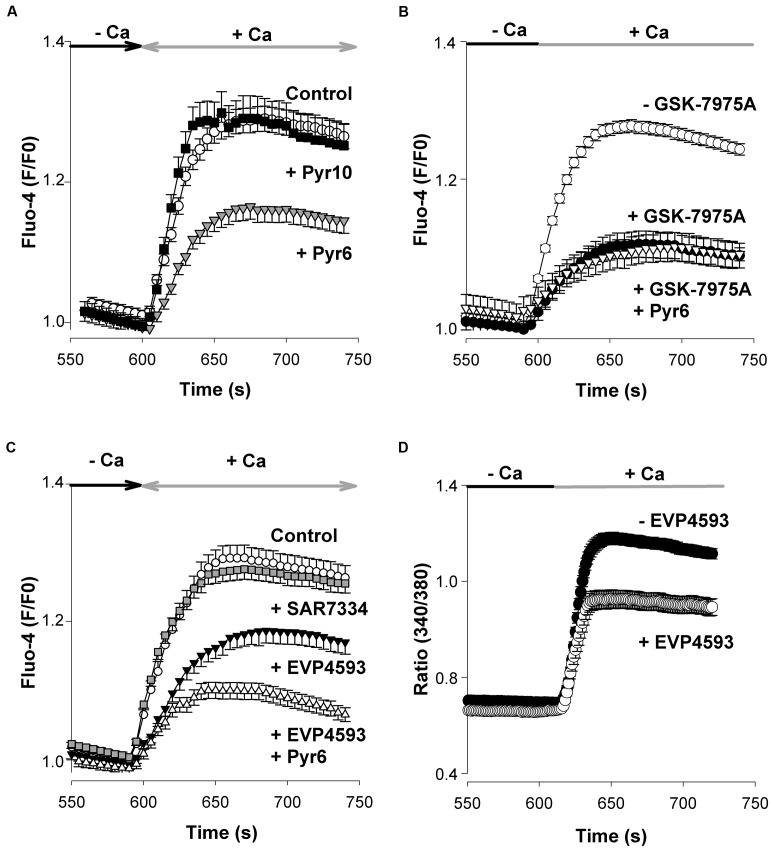
**Pyr6, GSK-7975A, and EVP4593 inhibit SOCE but not SAR7334. (A)** In these experiments Fluo-4 loaded cells were processed as in **Figure 2**. Pyr6 (gray triangles, *n* = 113 cells) or Pyr10 (filled squares, *n* = 96 cells; 1 μM) was added to the saline recording medium at time +60 s and remained present till the end of the experiment. Open circles: untreated (control) cells (*n* = 107). **(B)** Same experiments as in A except 5 μM GSK-7975A (filled circles, *n* = 118 cells), or GSK-7975A (5 μM) + Pyr6 (1 μM, open triangles, *n* = 73 cells, data from 2 cell cultures) were added to the saline at time +60 s prior to the application of Tg and remained present throughout the recording. Open circles: untreated (control) cells (*n* = 63 cells). **(C)** Same protocol as in A except SAR7334 (1 μM, gray squares, *n* = 134 cells), EVP4593 (1 μM, filled triangles, *n* = 112 cells), or EVP4593 (1 μM) + Pyr6 (1 μM; open triangles, *n* = 62 cells), were added at time +60 s. Open circles: untreated (control) cells (*n* = 117 cells). The joint application of EVP4593 (1 μM) + Pyr6 (1 μM) was tested on two cell cultures. None of the substance tested affected the Tg-evoked Ca^2+^ response (not shown). **(D)** EVP4593 depressed SOCE in RBL-2H3 cells. The effect of EVP4593 on native Orai channels from RBL-2H3 was tested by means of Fura-2 Ca^2+^ imaging experiments according to experimental procedures already described (Schleifer et al., 2012). EVP4593 (300 nM, open circles, *n* = 41 cells) was added to the saline at time +60 s prior to the application of Tg (200 nM) and remained present throughout the recording. Filled circles: Control (EVP4593 untreated) cells (*n* = 52). EVP4593 significantly reduced the peak amplitude of the native SOCE (*p* < 0.001, Student’s *t*-test). The graphs **(A–D)** show the last 200 s of the recording. Mean ± SEM.

To characterize the pharmacological profile of native SOCs, we took advantage of the recent development of blockers of Orai and TRPC channels. The compounds used are listed in **Table 2**. For instance, Pyr6 is a newly synthetized pyrazole compound that inhibits Orai1-mediated Ca^2+^ responses with a high potency (IC_50_ 0.49 μM; Schleifer et al., 2012). When applied at the concentration of 1 μM, Pyr6 reduced the peak amplitude of SOCE by nearly 50% (Student’s *t*-test, *p* < 0.01) (**Figure 3A**). In contrast, Pyr10, another pyrazole compound with a high potency to suppress TRPC3-mediated Ca^2+^ responses (Schleifer et al., 2012) had no effect when tested at the same concentration (1 μM) (**Figure 3A**). GSK-7975A, another Orai channel blocker, was used. It inhibits Orai1- and Orai3-mediated currents with an IC_50_ of 4.1 and 3.8 μM, respectively (Derler et al., 2013). As depicted in **Figure 3B**, 5 μM GSK-7975A reduced the peak amplitude of SOCE by ∼50% (filled circles, *p* < 0.001, Student’s *t*-test). Next, Pyr6 (1 μM), and GSK-7975A (5 μM) were applied together to see whether they had additive effects. As shown in **Figure 3B** (open triangles), Pyr6 did not strengthen the blockade of SOCE produced by GSK-7975A alone. These data show that native SOCs of cortical neurons from embryonic mice are sensitive to the Orai channels inhibitors Pyr6 and GSK-7975A, two compounds that have no additive effects.

**Table 2:** Alphabetical list of the Orai and TRPC channel blockers used in this study.

	Over-expression system				Native channels		
Compound	Cell type	Over-expressed proteins	IC_50_	Reference	Cell type	IC_50_	Reference
EVP4593	SK-N-SH neuroblastoma cells	TRPC1	No inhibition	Wu et al., 2011	Striatal neurons	SOC inhibited by 40% with 300 nM	Wu et al., 2011
GSK-7975A	HEK cells	CFP-Stim1/YFP-Orai1	4.1 μM	Derler et al., 2013	Rat basophilic leukemia (RBL-2H3) cells	0.8 μM	Derler et al., 2013
	HEK cells	CFP-Stim1/YFP-Orai3	3.8 μM	Derler et al., 2013			
	HEK cells	YFP-Orai3	no inhibition	Derler et al., 2013			
Pyr3	HEK cells	TRPC3	0.7 μM	Kiyonaka et al., 2009	DT40 B lymphocytes^1^	Complete inhibition with 1 μM	Kiyonaka et al., 2009
	HEK cells	YFP-TRPC3	0.54 μM	Schleifer et al., 2012	RBL-2H3 cells	0.54 μM	Schleifer et al., 2012
Pyr6	HEK cells	YFP-TRPC3	18.46 μM	Schleifer et al., 2012	RBL-2H3 cells	0.49 μM^2^ (Stim1/Orai1)	Schleifer et al., 2012
Pyr10	HEK cells	YFP-TRPC3	0.72 μM	Schleifer et al., 2012	RBL-2H3 cells	13.08 μM (Stim1/Orai1)	Schleifer et al., 2012
SAR7334	HEK cells	TRPC3	282 nM	Maier et al., 2015			
	HEK cells	TRPC6	9.5 nM	Maier et al., 2015	Isolated lungs	100 nM^3^	Maier et al., 2015
	HEK cells	TRPC7	226 nM	Maier et al., 2015			

*The main targets identified are given together as the IC_50_ values. This table also indicates if the experiments were conducted on over-expressed or on native channels. ^1^In DT40 B lymphocytes Ca oscillations are due to an entry of Ca^2+^ through TRPC3 channels. These oscillations are suppressed in the presence of 1 μM Pyr3. ^2^In RBL-2H3 cells, SOCE is mediated by Stim1/Orai1 (Hoth and Penner, 1992; Calloway et al., 2009). ^3^In this study the authors used a model of hypoxia-induced vasoconstriction that depends exclusively on TRPC6 activity. In isolated lungs, half maximal inhibition of hypoxia-induced vasoconstriction was achieved in the presence of 100 nM SAR7334.*

### Neuronal SOCE and Blockers of TRPC Channels

The exact role played by TRPC channels in the entry of Ca^2+^ through SOCE is still debated (Prakriya and Lewis, 2015). To clarify this issue, two recent TRPC channel blockers were used: SAR7334, which inhibits TRPC3-, TRPC6-, and TRPC7-mediated Ca^2+^ responses with IC_50_ values of 282, 9.5, and 226 nM (Maier et al., 2015), respectively, and EVP4593 which targets heteromeric but not homomeric TRPC1 channels (Wu et al., 2011). A concentration of 1 μM EVP4593 reduced the maximal amplitude of SOCE by ∼50% (filled triangles, *p* < 0.01, Student’s *t*-test) whereas the same concentration of SAR7334 had negligible effects (**Figure 3C**). In a set of experiments, the Orai channels blocker Pyr6 (1 μM) was added together with EVP4593 (1 μM). Under this condition, the inhibitory properties of Pyr6 and EVP4593 were only partially additive (open triangles, **Figure 3C**). Ca^2+^ imaging experiments were also conducted on RBL-2H3 cells where Orai1 channels are the core components of the native SOCs, without any contribution from TRPC1 channels (Schleifer et al., 2012). When added at 300 nM, EVP4593 significantly reduced the peak amplitude of SOCE in RBL-2H3 cells (*p* < 0.001, **Figure 3D**). This latter observation indicates that EVP4593 has the ability to depress the entry of Ca^2+^ through Orai channels.

### Neuronal SOCE and Extracellular Cations

**FIGURE 4:**
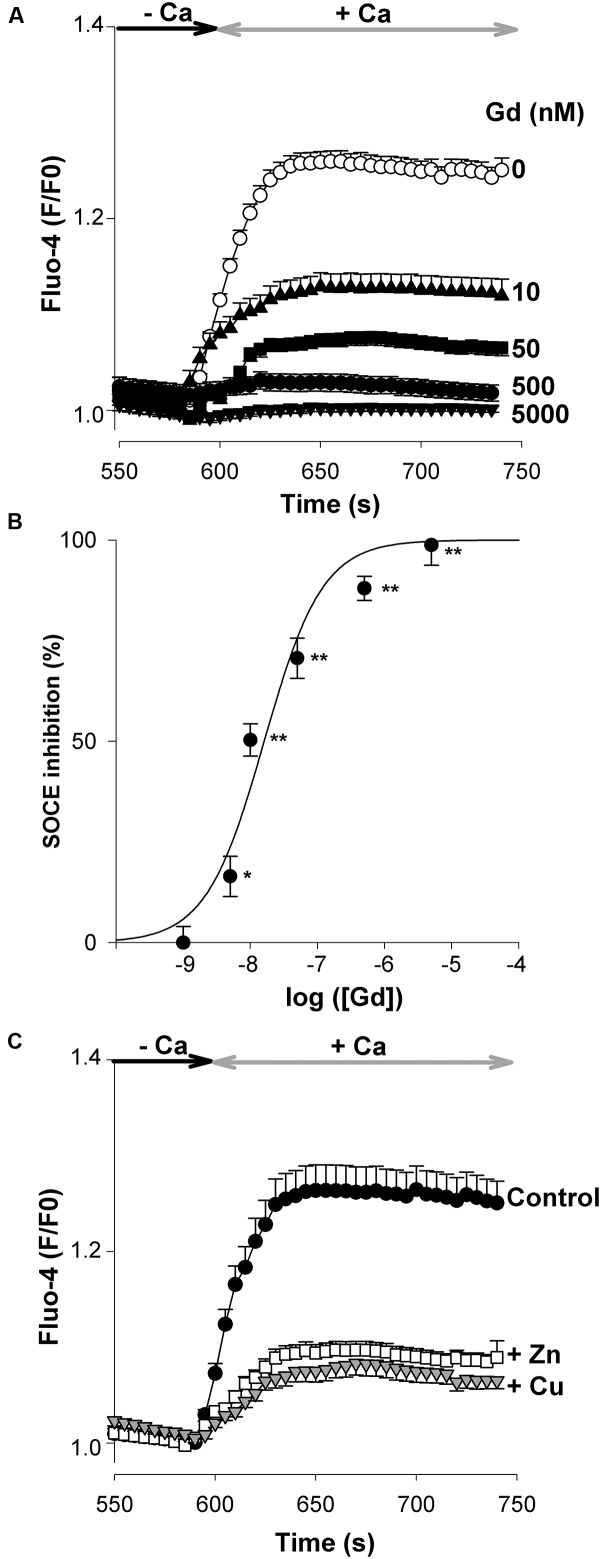
**Inhibition of SOCE by the external cations, Gd^3+^, Zn^2+^, and Cu^+/2+^.** The protocol used is the same as in **Figure 2**. **(A)** The Tg-evoked entry of Ca^2+^ was recorded in the absence (open circles, *n* = 102 cells) or presence of various concentrations of GdCl_3_ (filled symbols; 10 nM *n* = 144 cells; 50 nM *n* = 132 cells; 500 nM *n* = 112 cells; 5000 nM *n* = 67 cells). **(B)** dose-response curve showing the inhibition (%) of the peak amplitude of the SOCE as a function of the external concentration of GdCl_3_. ^∗^ and ^∗∗^, *p* < 0.05 and *p* < 0.01, respectively. **(C)** Inhibitory effect of 10 μM ZnCl_2_ (open squares, *n* = 129 cells) and 10 μM CuCl_2_ (gray triangles, *n* = 115 cells) on SOCE. Filled circles: untreated (control) cells (*n* = 101 cells). The graphs **(A,C)** show the last 200 s of the recording. Mean ± SEM.

Lanthanides like gadolinium (Gd^3+^) ions have been employed as blockers of SOCs. In the murine brain, neuronal SOCE is highly sensitive to Gd^3+^ (**Figure 4A**), with the peak amplitude of SOCE being reduced by ∼50% (*p* < 0.01, Student’s *t*-test) with ∼10 nM Gd^3+^ (**Figure 4B**). In some brain areas like the hippocampus and the cortex, neuronal activity can be associated with the release into the synaptic cleft of essential cations like zinc (Zn^2+^) and copper (Cu^+/2+^) exerting neuromodulatory actions by acting on diverse neurotransmitter receptors and channels (Bush, 2003). We next addressed the question of the effects of these two cations on native SOCE. As illustrated in **Figure 4C**, a concentration of 10 μM ZnCl_2_ (open squares) or CuCl_2_ (gray triangles) depressed SOCE strongly and equally.

### Neuronal SOCE and iPLA_2_

**FIGURE 5:**
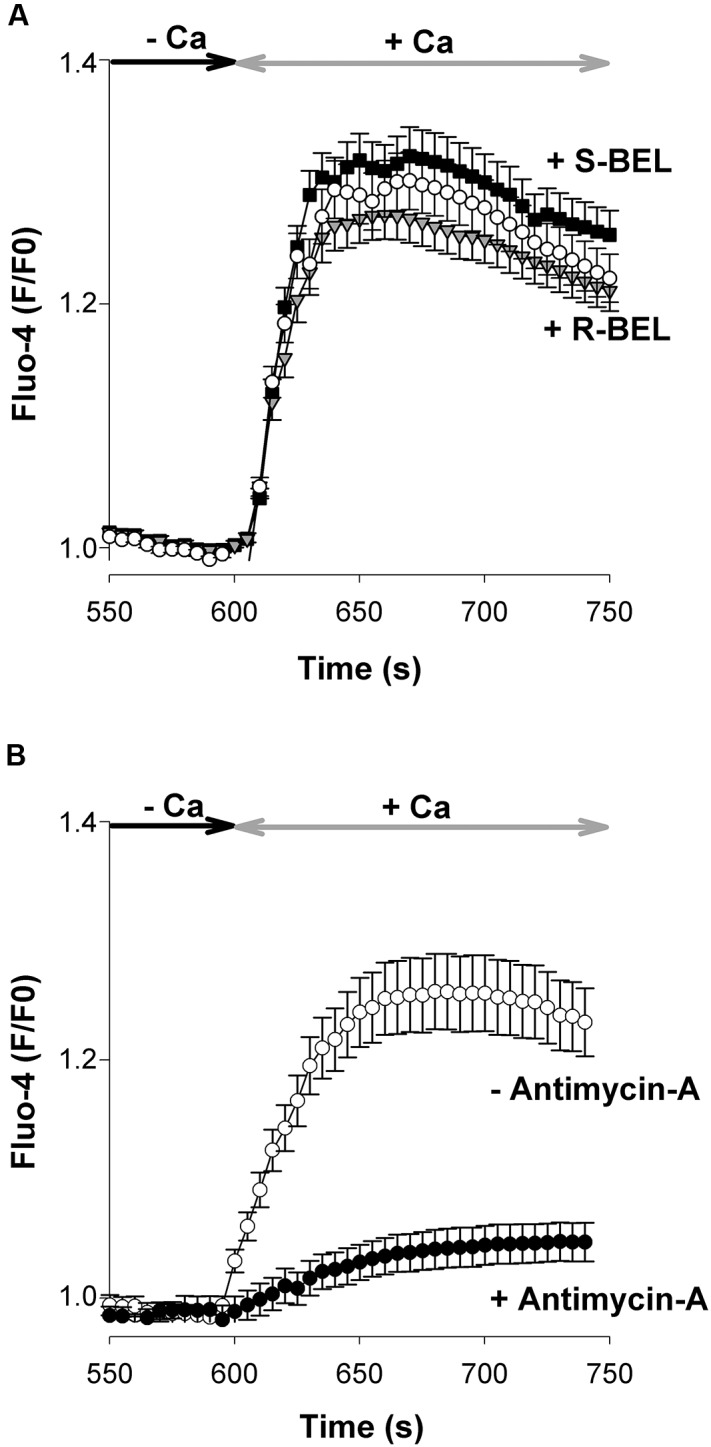
**Mitochondria but not iPLA_2_ regulate SOCE in cultured cortical neurons. (A)** In these experiments cells were pretreated with (S)-BEL (filled squares, *n* = 144 cells) or (R)-BEL (gray triangles, *n* = 167 cells; 5 μM for 30–40 min at 37°C) before the beginning of the recording. None of these iPLA_2_ inhibitors significantly affected the neuronal SOCE. Open circles: control (untreated) neurons. Mean ± SEM. **(B)** In order to check the contribution of mitochondria in the development of neuronal SOCE, cells kept in a Ca^2+^-free saline were first challenged with Tg (200 nM) that was applied without (open circles, *n* = 101 cells) or with 5 μg/mL Antimycin A (filled circles, *n* = 129 cells). Tg and Antimycin-A were no longer present during the re-admission of Ca^2+^ (2 mM). Mean ± SEM. In these graphs only the Fluo-4 signals recorded during the re-admission of external Ca^2+^ are shown.

Several hypotheses have been put forward to explain how the release of Ca^2+^ from the ER could activate channels located in the PM. For instance, Ca^2+^ store depletion would cause the release of a calcium influx factor (CIF) stimulating the activity of the Ca^2+^-independent phospholipase A_2_ (iPLA_2_) followed by the activation of SOCs (Smani et al., 2003; Csutora et al., 2006). In order to verify the contribution of iPLA_2_, experiments were conducted with 2 enantiomers of bromoenol-lactone (BEL), an iPLA_2_ inhibitor: (S)-BEL and (R)-BEL, which depress the activity of iPLA_2_β and iPLA_2_γ, respectively. In smooth muscle cells, (S)-BEL, but not (R)-BEL, depresses SOCE with an IC_50_ of ∼3 μM (Csutora et al., 2006). When tested at a concentration of 5 μM, none of these compounds affected the neuronal SOCE (**Figure 5A**).

### Mitochondrial Regulation of SOCs

Mitochondria are physiological regulators shaping Ca^2+^ signals (Pizzo and Pozzan, 2007), notably by buffering Ca^2+^ ions delivered by plasmalemmal Ca^2+^ channels, including SOCs (Hoth et al., 1997). It is thought that Ca^2+^ entering through activated SOCs inhibits their activity. Consequently, impairing the Ca^2+^ buffering property of mitochondria depresses SOCE (Parekh, 2003). E13 cortical neurons were incubated with antimycin-A, an inhibitor of the mitochondrial electron transport chain. This provoked a strong reduction of SOCE (**Figure 5B**), which confirms the ability of these organelles to control the Ca^2+^ signals generated by SOCs.

### Resting Ca^2+^ Entry

**FIGURE 6:**
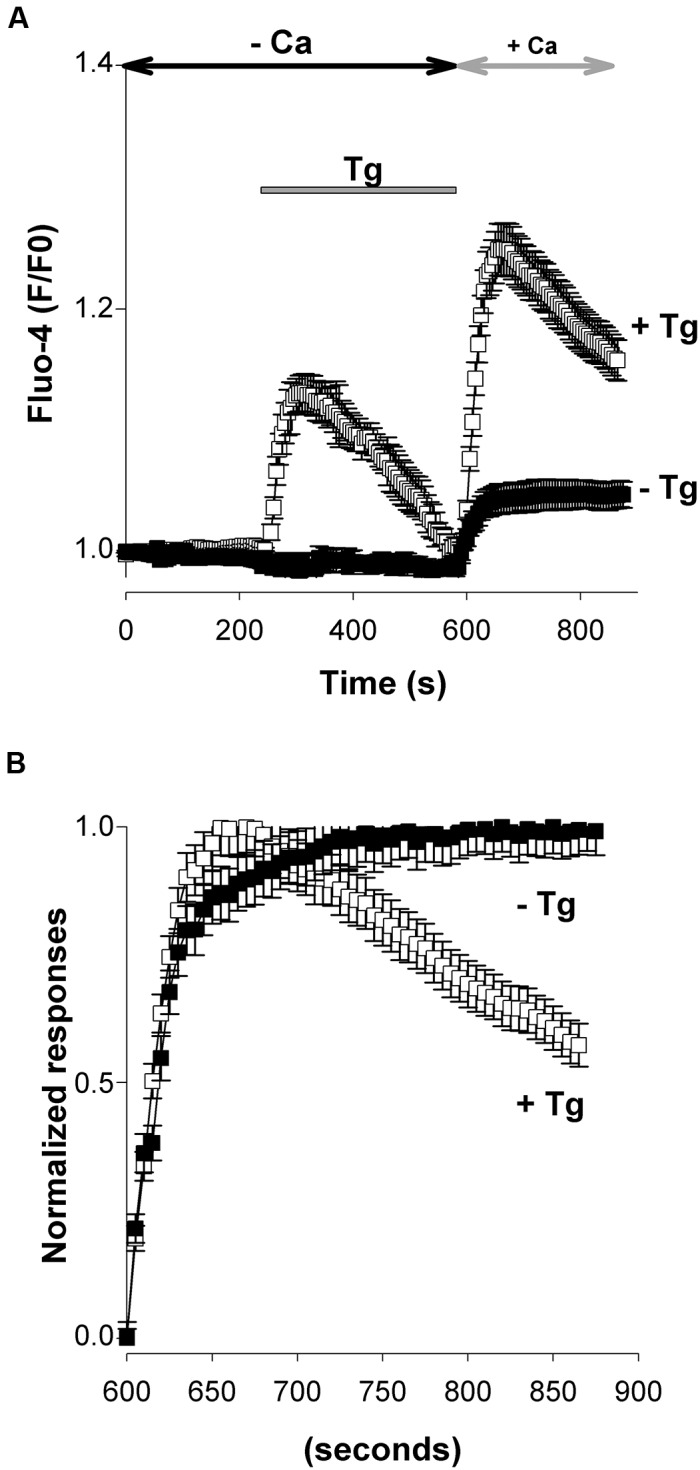
**Resting Ca^2+^ entry and SOCE. (A)** Same protocol as in **Figure 2**. Some neurons were challenged with Tg (200 nM, open squares, *n* = 101 cells) to elicit a SOCE. In another set of experiments, Tg was omitted (filled squares, *n* = 139 cells). **(B)** Same data as in (**A**; from time +600 to 875 s) after the re-admission of Ca^2+^ (2 mM) into the external recording saline. Signals have been normalized. Mean ± SEM.

Additional Ca^2+^ imaging experiments were conducted but without applying thapsigargin. Under this condition, the re-introduction of Ca^2+^ into the external recording saline was also followed by an elevation of the Fluo-4 fluorescence amounting to 15–20% (filled squares, *n* = 139 cells) of the peak amplitude of the thapsigargin-activated Ca^2+^ entry (SOCE, open squares) (**Figure 6A**). Another key feature of this resting Ca^2+^ entry is its time course. This is illustrated in **Figure 6B** where both signals (SOCE and resting Ca^2+^ entry) have been scaled up. The neuronal SOCE peaked and then slowly declined over time (a run-down which most probably reflects the Ca^2+^-dependent inactivation of the SOCs) whereas the resting Ca^2+^ entry gave rise to a sustained response (**Figure 6B**, filled squares). In addition, this resting Ca^2+^ entry was insensitive to various Orai blockers (10 nM Gd^3+^, *n* = 83 cells; 1 μM Pyr6, *n* = 89 cells; 1 μM EVP4593, *n* = 59 cells; not shown), tested at concentrations that depressed SOCE by ∼50%. It was also insensitive to the dihydropyridine nifedipine blocking L-type Ca^2+^ channels (10 μM, *n* = 67 cells; not shown). Therefore, the resting Ca^2+^ entry and SOCE develop through channels possessing distinct pharmacological and kinetic properties. Overall, these data indicate that SOCE is mediated by Orai2 channels, the only Orai isoform found in this tissue at that embryonic age, whereas the passive Ca^2+^ entry seems to develop independently of Orai channels via dihydropyridine-insensitive channels.

## Discussion

This study was initiated to further advance the molecular and pharmacological characterization of native SOCs of embryonic (E13) cortical neurons from murine brains. To this aim, the mRNA expression profile of putative SOC components like STIM1-2 and Orai1-3 was assessed by qPCR. In primary cultures of E13 cortical neurons the qPCR analysis failed to detect Orai1 transcripts whereas Orai3 transcripts were only barely detected. Additionally, Orai1 was also lacking and low levels of Orai3 transcripts were found in the immature cortex. Overall, Orai2 was by far the main Orai mRNA present in the immature E13 cortex and in primary cultures of E13 cortical neurons. STIM1 and STIM2 transcripts were also found in both preparations with STIM2 being the predominant STIM isoform. These results are in line with a previous study showing that STIM2 is the predominant STIM isoform in cultured cortical neurons from E19 rat brain (Gruszczynska-Biegala et al., 2011). Orai1 is also not found in cultured E18 hippocampal neurons dissociated from mice (Gruszczynska-Biegala et al., 2011) but is detected in cultured E19 cortical neurons from rat brain (Berna-Erro et al., 2009). On the other hand, Orai2 is the only Orai isoform in cultured hippocampal neurons from E18 mice (Berna-Erro et al., 2009). Experiments conducted on Orai1 knock-out (Orai1^-/-^) and STIM1 knock-out (STIM1^-/-^) mice showed that none of these actors contribute to SOCE in the brain (Berna-Erro et al., 2009). Altogether, our qPCR analysis indicates that, in the immature cortex, the principal STIM and Orai transcripts are STIM2 and Orai2. Preliminary immunocytochemical experiments confirmed the expression of Stim 2 and Orai 2 in cultured cortical neurons (**Supplementary Figure S1**). The accumulated experimental data, whether derived from studies on native neuronal SOCs or from studies on knock-out mice, highlight the central role played by STIM2 and Orai2 in neuronal SOC activity.

The pharmacological analysis of native SOCs of E13 cortical neurons shows that they were sensitive to the Orai channel blockers Pyr6 and GSK-7975A. Pyr3, a Orai and TRPC3 channel blocker, was also shown to inhibit native SOCs (Gibon et al., 2010). These channels were neither affected by the TRPC3-channel blocker Pyr10 nor by the TRPC3, TRPC6, and TRPC7 channel inhibitor SAR7334. Interestingly, SOCE was depressed by EVP4593, a compound originally described as a blocker of heteromeric TRPC1 channels but having no effect on homomeric TRPC1 channels (Wu et al., 2011), implying that TRPC1 is not a target of EVP4593. We further advance the pharmacological description of EVP5493 action by showing that it depressed native SOCs of RBL-2H3 cells which consist of Orai channels. Interestingly, Pyr6 (described as an Orai1 blocker) and GSK-7975A (described as an Orai3 blocker, see **Table 2** for references) exerted the same inhibitory action on native neuronal SOCs whereas Orai2 was the sole Orai channel (at the mRNA level). Furthermore, the effects of Pyr6 and GSK-7975A were not additive, indicating they act on a common target. However, a joint addition of Pyr6 + EVP4593, tested at concentrations reducing the peak SOCE by half, did not fully block SOCE, their effects being partially additive. Based on this finding it is tempting to propose that TRPC1, together with Orai proteins, is involved in neuronal SOC. Of note, TRPC1, at the mRNA level, is the most abundant TRPC found in the E13 murine brain and cortex (Boisseau et al., 2009). However, this view derived from a pharmacological analysis is hypothetical and it remains to be shown whether TRPC1 assembles with Orai2 to form neuronal SOCs.

Store-operated channels are highly sensitive to lanthanides like gadolinium (Gd^3+^). These cations can thus be used to discriminate SOCs from other Ca^2+^-conducting channels such as TRPC and voltage-gated Ca^2+^ channels which require micromolar amounts of Gd^3+^ for blockade. In the embryonic brain, native SOCs displayed a high sensitivity to Gd^3+^ which abolishes SOCE with an IC_50_ of ∼10 nM. Of note, Gd^3+^ ions block CRAC (a subtype of SOCs formed by Orai proteins) with IC_50_ values of 28 and 50 nM in thymocytes and drosophila S2 cells, respectively (Ross and Cahalan, 1995; Yeromin et al., 2004). This high sensitivity of neuronal SOCs to Gd^3+^ further suggests that Orai, and not TRPC, channels contribute to SOCE in this cell type. SOCs of embryonic cortical neurons were depressed by Zn^2+^ and Cu^+/2+^, two essential cations that can be released synaptically upon neuronal stimulation (Bush, 2003). In our experiments, 10 μM ZnCl_2_ (or CuCl_2_) strongly depresses SOCE. This indicates that synaptic SOCs are likely to be down-regulated at active synapses releasing Zn^2+^ and/or Cu^+/2+^.

The contribution of iPLA_2_ in SOCE has been shown for SOCs composed of STIM1 and Orai1 in smooth muscle cells and RBL cells (Bolotina, 2008). However, in embryonic cortical neurons, the pharmacological inhibition of iPLA_2_β [with (S)-BEL] or iPLA_2_γ [with (R)-BEL] was not associated with any impairment of SOCE. Of note, BEL blocks Ca^2+^ entry through voltage-gated Ca^2+^ channels and TRPC1, TRPC5, and TRPC6 channels with IC_50_ values of 7–8 μM (Chakraborty et al., 2011). Another interesting property of native neuronal SOCs is their regulation by mitochondria. Indeed, Antimycin A, which depolarizes mitochondria and prevents the mitochondrial Ca^2+^ uptake, strongly depressed the entry of Ca^2+^ though SOCs. These channels, like other types of Ca^2+^ channels, are subject to a Ca^2+^-dependent inactivation process which down regulates the influx of Ca^2+^. By buffering Ca^2+^ ions entering via SOCs, mitochondria modulate Ca^2+^ microdomains, precluding, or minimizing the Ca^2+^-dependent inactivation of SOCs, permitting larger influx of Ca^2+^ to occur (Parekh, 2003).

## Conclusion

Our data indicate that Orai2 is a critical pore component of native neuronal SOCs in the immature cortex. They are sensitive to various Orai blockers like Pyr6 and GSK-7975A. These channels are also blocked by EVP4593, and the cations Gd^3+^, Zn^2+^, and Cu^+/2+^. Gd^3+^ cations are by far the most potent neuronal SOCs blocker tested. Of note, none of the Orai channel blockers used (Pyr6, GSK-7975A) can be employed to discriminate native Orai channels since they inhibit all types of Orai channels. Mitochondria are controlling SOCE which seems to develop without the requirement of iPLA_2_ activity. On the other hand, the passive entry of Ca^2+^ exhibit distinct pharmacological and kinetics profiles indicating that they deliver Ca^2+^ to neurons via channels distinct from SOCs. The presence of functional SOCs at the beginning of cortico-genesis suggests that this Ca^2+^ route is likely to play a role in the formation of the brain cortex.

## Author Contributions

SC performed experiments and analyzed data. LJ performed experiments and analyzed data. NS performed experiments and analyzed data. MC performed experiments and analyzed data. KG contributed new reagents. AB designed the study, performed experiments, analyzed data and wrote the manuscript.

## Conflict of Interest Statement

The authors declare that the research was conducted in the absence of any commercial or financial relationships that could be construed as a potential conflict of interest.

## Supplementary Material

FIGURE S1 **Detection of Stim1, Stim2, Orai1, Orai2, Orai3 by immunocytochemistry**.
