# Corrigendum: Pharmacological Characterization of the Native Store-Operated Calcium Channels of Cortical Neurons from Embryonic Mouse Brain

**DOI:** 10.3389/fphar.2017.00152

**Published:** 2017-03-22

**Authors:** Sylvain Chauvet, Louis Jarvis, Mireille Chevallet, Niroj Shrestha, Klaus Groschner, Alexandre Bouron

**Affiliations:** ^1^UMR CNRS 5249, Commissariat à l'Énergie Atomique et aux Énergies AlternativesGrenoble, France; ^2^Université Grenoble AlpesGrenoble, France; ^3^Institut de Biosciences et Biotechnologies de Grenoble – Laboratoire de Chimie et Biologie des MétauxGrenoble, France; ^4^Institute of Biophysics, Medical University of GrazGraz, Austria

**Keywords:** store-operated channels, calcium, Orai channels, neurons, cortex

**Missing Funding**

In the original article, we neglected to include the funder **Austrian Science Fund FWF**, **Grant Number W1226** to **Niroj Shrestha**. The authors apologize for this error and state that this does not change the scientific conclusions of the article in any way.

## Funding

This work was financially supported by the Centre National de la Recherche Scientifique (France) and the Austrian Science Fund FWF (Grant Number W1226 to NS).

### Conflict of interest statement

The authors declare that the research was conducted in the absence of any commercial or financial relationships that could be construed as a potential conflict of interest.

