# Supplementary figures and images for: Pharmacological Characterization of the Native Store-Operated Calcium Channels of Cortical Neurons from Embryonic Mouse Brain
Source: Front Pharmacol. 2016 Dec 12;7:486. doi: 10.3389/fphar.2016.00486 (PMC5149554; doi:10.3389/fphar.2016.00486)

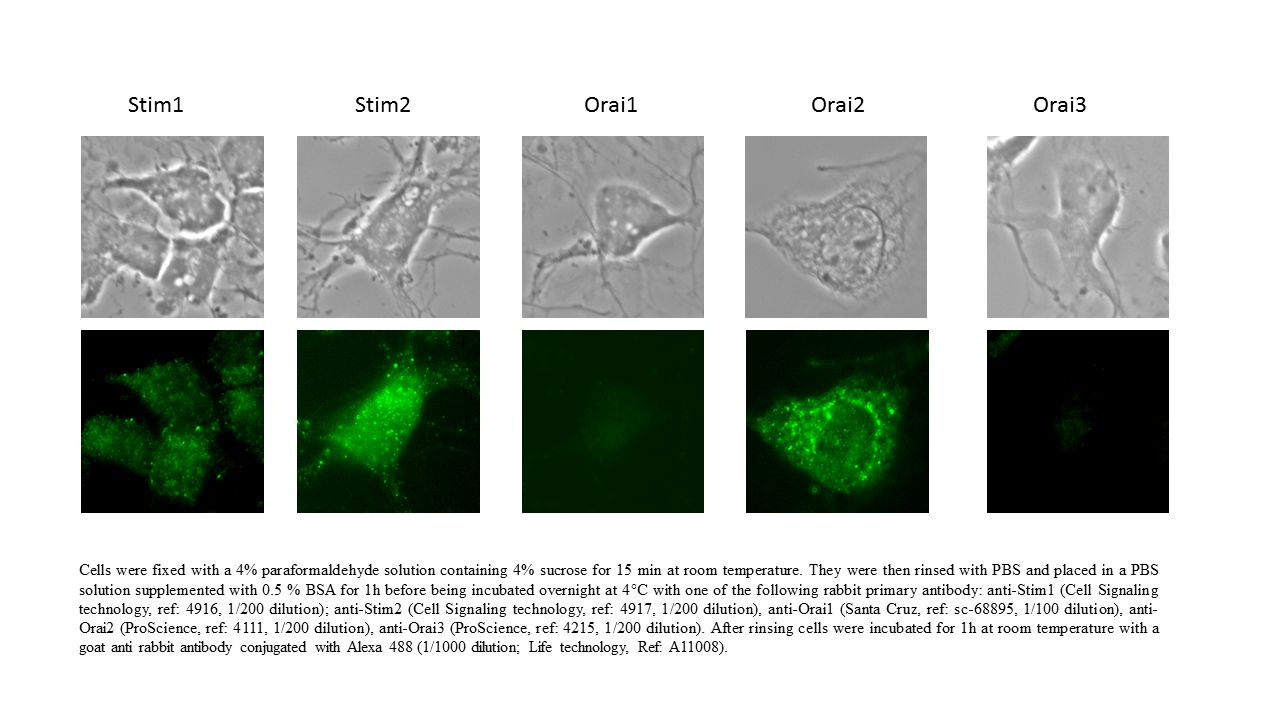

Supplement: FIGURE S1 — Detection of Stim1, Stim2, Orai1, Orai2, Orai3 by immunocytochemistry. [file Image_1.tif]
